# Looking through the patient lens – Improving best practice for young people with juvenile idiopathic arthritis transitioning into adult care

**DOI:** 10.1186/s40064-015-0888-8

**Published:** 2015-03-05

**Authors:** Samantha Howland, Kay Fisher

**Affiliations:** Pfizer Ltd, Walton Oaks, Dorking Road, Tadworth, Surrey KT20 7NS UK; Experience Engineers, Chalfont St Peter, Bucks, UK

**Keywords:** Paediatric rheumatology, Juvenile idiopathic arthritis, JIA, Transition, Experience, Adolescent

## Abstract

We describe a qualitative study to establish the emerging needs of young people with juvenile idiopathic arthritis (JIA) as they go through the transition process, identifying which elements are valued and where support gaps exist.

Qualitative interviews with healthcare professionals, young people with JIA and their parents explored the lived experience of transition to care in an adult rheumatology clinic. Perspectives of the experience and reflections of the process of transitioning were captured along with the young people’s views of optimal support.

Service provision in the clinical environment varied. Service design for this particular patient group has an impact on how young people optimise management of, and engagement with, their condition during young adulthood. Two specific themes emerged that had the greatest impact on defining a positive user experience of transitioning care: tailored service provision within the clinical environment and support for those living with JIA beyond the clinic doors (we have termed these the lived experience). Factors of importance to young people with JIA were grouped into key domains, namely: day-to-day life with JIA, emotional and developmental factors and a desire for independence.

The young people and healthcare professionals interviewed in this small qualitative study highlighted some common themes considered critical in the development and organisation of an excellent care pathway from paediatric to adult healthcare. Aligning the clinical process with young people’s individual needs and lifestyle creates stronger beginnings in adult care.

## Introduction

Putting the patient at the centre of the healthcare strategy sits at the heart of care provision in the UK. This is never more important than in the consideration of young people with chronic conditions as they transition from paediatric services into adult healthcare.

During adolescence and young adulthood, it is well accepted that individuals with chronic conditions have the added complexity of managing their medical needs alongside their rapid physical, social, vocational and psychological growth, and their drive towards independence (Tattersall & McDonagh 2010; World Health Organization 2014; World Health Organization 2012). The NHS Clinical Reference Group for Paediatric Medicine (NHS Clinical Reference Group E03 – Paediatric Medicine 2013) as well as the UK Department of Health (Department of Health 2011) and the British Society for Paediatric and Adolescent Rheumatology (Davies et al. 2010) place importance on the development of appropriate transitional care as young people move into the adult service. However, this often presents significant challenges for many young people, their families and healthcare professionals (Coulson et al. 2014).

This article reports work undertaken in 2013 in the UK with healthcare professionals, young people with juvenile idiopathic arthritis (JIA) who take/or have taken a biologic medicine, and family members. A qualitative study was designed to establish the emerging needs of young people with JIA and to capture their existing experience in order to understand which components of healthcare they truly value, and why.

## Methods

Two rheumatologists (one adult and one paediatric), four specialist nurses and a physiotherapist who work with adolescents, plus six young people with JIA (aged 16–18 years), and five parents were interviewed in this study. Young people were interviewed individually, and in five of the six cases we went on to conduct paired interviews with the parent/carer present. At the time of the interviews the young people was in various stages of their transitional care into adult rheumatology. This was designed to gain real time insights around their immediate needs.

The interviews used a qualitative technique (interpretive phenomenonological analysis) (Smith et al. 2009) rooted in exploring the ‘lived experience’ of the young person and those around them. The sample sizes, although small, were sufficient to build themes of needs, which represent the entire sample. The interviews were recorded and analysed for common themes.

All of the interviews were conducted in the young people’s homes rather than a clinical setting, which enabled closer observation of the impact of JIA on their lives. Interviews lasted approximately 60 minutes. Young people were given time to discuss what mattered to them, which enabled us to review the findings using patient-based phenomena. Participants were selected based on access to a variety of transitional care models so that we could assess the value of each to the overall experience of care.

Young people’s perspectives on their experience and their changing support needs were captured, along with their view of optimal support. Insights were gained into aspects of living with JIA as a teenager and linked to areas of demand on the healthcare system generally, and during the transition process specifically.

## Results

The young people in this study had all experienced different models of care and our interpretation of these models will be discussed later.

Feedback from the interviews identified key insights, from both the young person and professional perspective, about aspects of transitional care. Interviews with young people with and without a parent provided additional insight into the dynamics of the familial relationships in these cases.

Two specific themes emerged, which healthcare professionals and young people told us have the greatest impact on defining a positive patient experience:

- Service provision within the clinical environment
- Life with JIA outside of the clinical environment.

### Service provision within the clinical environment

Bearing in mind the continuing requirement of care for individuals with JIA moving into adulthood, some key inflection points were identified in the user experience, where variables might affect engagement of young people with their condition, and may therefore influence outcomes. In particular, our respondents recognised the benefits of the following:

#### Familiarisation

Young people who had been given an opportunity for familiarisation with the adult clinic felt less apprehensive and more comfortable about the transition process. Examples of familiarisation included a visit to the adult clinic with a nurse and an opportunity to meet with the consultant who would be conducting their first adult consultation. Whilst young people recognise the need to become more independent around their disease management, they are better able to focus on their disease needs at clinic if their anxieties around new people and a new place are reduced.

#### Access to specialist nurses

Not all clinics provide access to an appointment with a specialist nurse adjacent to the first appointment at the adult clinic. Where this is designed into the service, young people have a more immediate connection, and feel more secure in their ability to self-manage, knowing they have support on hand.

#### Benchmarking patient knowledge

Young people vary in their knowledge and understanding of their disease and its optimum management. Some have relied on parents or carers for this; some have taken a more active interest in their treatment plan. Participants had often developed a working partnership with their paediatric consultant over time and had made adjustments to treatment plans, giving them a sense of control over their disease and their lives. It is important to young people that their knowledge and understanding is clarified and documented as they move into adult care. Where this benchmarking was built into the transition process, they felt better understood, and it gave them an opportunity to build knowledge about their treatment and the benefits of its management.

**Figure 1:**
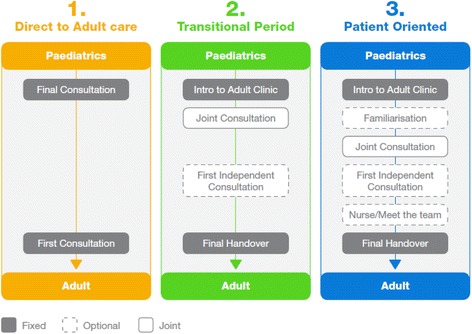
**Strategies identified in this study to transition patients from paediatric to adult rheumatology care.**

The different ‘transition’ strategies in the centres involved could be broadly grouped into three types of service, as illustrated in Figure 1. Unless there were geographical difficulties, young people preferred the care model that provided opportunities for familiarisation with both the healthcare team and adult clinic itself. They also benefited from more clarity around the transition process; none of the young people we spoke to was completely clear on some of the key facts, such as who they could expect to see; when they could expect to see them; how long the process would take; the type, duration and frequency of consultations they would be attending during the process and beyond; and when the transition process would be complete.

Whilst most young people transfer into adult care around the age of 16 years, this varies depending on the centre and the individual’s social situation, personality and history of engagement with treatment. Successful transition requires engagement with all aspects of a young person’s history and current status.

All of the individuals we spoke to in this study reported gaps in the continuum of care, not in terms of clinical management of the condition, but in terms of their changing needs as young adults. Thus the ‘patient-oriented care’ model shown in Figure 1 was more likely to capture issues specific to young people which, if not addressed, could impact their disease management going forward. Most clinicians interviewed in the study recognised that this ‘patient-oriented care’ model would better serve the needs of young people and would also impact on outcomes, concordance and engagement.

### The Lived experience for young people in transitional care

The importance of the clinical team engaging with, and understanding, the lives of young people is critical during the transition process. This provides clinicians and young people with the opportunity to establish a healthy approach to managing the condition day to day, but also to manage flare-ups of JIA, which lead to an additional burden both on the hospital resources, and the patient. The transition period often coincides with one of the most stressful period of a young person’s life to date; they are faced with new situations and lifestyle changes within which their JIA needs to be accommodated. If their clinical team is also changing due to their physiological development into adulthood, this leaves a huge vacuum of support, and a lost opportunity to put these young people in control.

During our interviews with young people it became clear that their main concerns were not with their clinical outcomes per se, but the degree to which they could successfully manage their daily lives. Clearly the two (clinical outcomes and patient-reported outcomes) are linked, but we observed clear opportunities for this link to be strengthened to the benefit of both patient and clinician. Support needs in transition are demonstrably different from those of patients in paediatric care, and those who are established in adult care.

The perspective of the young people and parents and their areas of focus differed to those of the healthcare professionals that we interviewed. Descriptions and perceptions of good support from young people and families focused on ‘accommodating’ JIA into their lives, the changing paradigm of growing independence and the need for support as required. For healthcare professionals, emphasis was placed on clinical outcomes, concordance with prescribed regimens and appointment attendance. This came clearly into focus when we reviewed the issues articulated by patients as the things that mattered most to them. The examples in Table 1 represent themes that emerged during the interviews, which are representative of the whole sample.

**Table 1:** **Factors of importance to patients (a summation of patient perspectives)**

Day-to-day life with JIA	I want to find the right balance of treatment that fits in with my life
	I want to be able to manage my new responsibilities such as a new job
	I want to be able to deal with the physical limitations my condition places on me, such as carrying books
	I want to be in control of my condition whilst at university
Emotional and developmental factors	I need to find the right language for me when discussing my condition with my boyfriend/ girlfriend/ friends
	I am concerned about flare-ups during the summer when my body is on show
	I need coping mechanisms to be able to keep up with my friends and our social lives
Seeking independence	I want to put everything in place to facilitate my travelling plans
	I want to be able to manage without my parents
	I want to prove to my parents that I can manage my condition successfully
	I want a portfolio of options to be able to manage pain or flares when this occurs, each one to be used depending on my circumstances

## Discussion

The young people and healthcare professionals in this small qualitative study highlighted some common themes that they considered critical in the development and organisation of an excellent transitional care pathway. The themes are similar to those previously identified in other research in transitional care in chronic conditions (Crowley et al. 2011; Hilderson et al. 2010). It seems there are opportunities for change in delivering patient-centric care for young people with JIA. We have summarised the suggestions from the interviews into two categories: (a) service design and (b) young person engagement.

### Service design

Young people with JIA seek an appreciation of how their care and clinic visits will be different when they transition into adult rheumatology centres (Tattersall 2012). Regardless of the approach adopted for managing transition, explanations of what to expect, and from which healthcare providers, give young people confidence that the new centre will meet their individual needs.

Visits to the adult clinic and a chance to meet their new healthcare team prior to their first appointment enable young people to take ownership of the transition process by increasing familiarity with the new clinic. As with transition services in other areas of medicine, access to care, frequency and duration of appointments may be notably different in adult care compared with paediatric services (Lugasi et al. 2011). A number of topics were raised (such as fitting appointments around college timetables) that have led us to believe that flexibility is crucial during transition. The nature of the consultation with young people needs a different focus to other adults in clinic and hence requires careful consideration and planning.

The importance of access to specialist nurses was raised in all of our interviews. Whilst the young people interviewed were all receiving biologic therapies, this need was driven from a holistic care point of view rather than reference to any specific therapy.

### Young Person engagement

Interviewees raised concern about their limited assertiveness in asking their new clinical team for clarification or in raising other issues. Familiarisation with staff from the start, and provision of accessible information around key clinical terms and clinical team members was more likely to break down these barriers and encourage young people to come forward.

Transition also provides clinicians with an opportunity to re-engage young people, and thus put them in control of managing their condition and potentially optimise outcomes. Early appointments with transitioning patients provide clinicians with an opportunity not only to assess a young person’s condition, but also to assess their understanding of their condition, level of engagement in their healthcare, and their goals, objectives and psychosocial health. These findings corroborate with other work from the UK reporting focused group discussions and questionnaire based research with young people with JIA (Shaw et al. 2004; Shaw et al. 2007). ‘Life factors’ such as family and social situations should not be underestimated.

The young people in this study emphasised the importance of making their healthcare ‘fit in’ with their daily lives. People of this age are likely to engage with their treatment to a greater extent if the conversations with healthcare professionals are focused on the ‘here and now’ rather than their specific disease parameters and the exchange of clinical facts. The alignment of activities in and out of clinic is crucial to optimise a young person’s lived experience during transition into adult care.

To encourage ownership and engagement in health management beyond the clinic doors, we suggest particular attention be paid to exploring with young people where day-to-day healthcare (such as physiotherapy and medicines) fits into their life plan.

In summary, our study has enabled us to offer a perspective about what ‘success’ looks like for individuals who are in transitional care and who are managing their JIA condition into their young adult lives. The context of adolescent health is important to consider in any discussion regarding health transitions and aspects such as the lived experience is fundamental to adolescent health irrespective of condition whether rheumatic or otherwise. Further exploration is still warranted through wider discussion with clinical teams and young people, in both paediatric and adult settings, to further tailor the services available.

## Conclusions

In this small study, we have reviewed how young people define success, both from a service delivery perspective and also how improvements in this area might optimise their day-to-day management and control of their condition outside of the clinic. By consciously aligning the clinical process with young person’s own individual needs and lifestyle, we can create strong beginnings in adult care, which may positively influence patient reported-outcomes further into treatment.
